# Poly(2‐oxazoline)s with a 2,2′‐Iminodiacetate End Group Inhibit and Stabilize Laccase

**DOI:** 10.1002/cbic.201900561

**Published:** 2019-12-13

**Authors:** Montasser Hijazi, Esra Türkmen, Joerg C. Tiller

**Affiliations:** ^1^ Department of Bio- and Chemical Engineering TU Dortmund Emil-Figge-Strasse 66 44227 Dortmund Germany

**Keywords:** enzyme catalysis, inhibitors, laccases, polymers, poly(2-oxazoline)s

## Abstract

Poly(2‐oxazoline)s (POxs) with 2,2′‐iminodiacetate (IDA) end groups were investigated as inhibitors for laccase. The polymers with the IDA end groups are reversible, competitive inhibitors for this enzyme. The IC_50_ values were found to be in a range of 1–3 mm. Compared with IDA alone, the activity was increased by a factor of more than 30; thus indicating that attaching a polymer chain to an inhibitor can already improve the activity of the former. The enzyme activity drops to practically zero upon increasing the concentration of the most active telechelic inhibitor, IDA‐PEtOx_30_‐IDA (PEtOx: poly(2‐ethyl‐2‐oxazoline)), from 5 to 8 mm. This unusual behavior was investigated by means of dynamic light scattering, which showed specific aggregation above 5 mm. Furthermore, the laccase could be stabilized in the presence of POx‐IDA, upon addition at a concentration of 20 mm and higher. Whereas laccase becomes completely inactive at room temperature after one week, the stabilized laccase is fully active for at least a month in aqueous solution.

## Graphical abstract

**Bulking up**: Poly(2‐oxazoline)s (POx) with iminodiacetate (IDA) end groups competitively inhibit the enzyme laccase. A telechelic IDA‐POx‐IDA derivative shifts the inhibition mechanism at higher concentrations to become a dead‐end inhibitor. Simultaneously, the polymers stabilize the enzyme to preserve its activity, even for 28 days at room temperature.

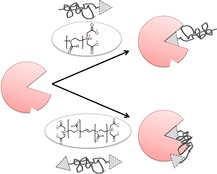

## Introduction

The inhibition of enzymes is an important topic for controlling biocatalytic processes relevant in medicine, bioanalytics, and agriculture. Most enzyme inhibitors are small molecules that interact with enzymes in several ways. Improving the activity of such molecules is a great benefit because lower inhibitor concentrations will minimize side effects and environmental pollution. Typically, such inhibitors are improved by chemical modification to increase binding to the active site of the respective enzyme and to increase specificity.^1^, ^2^

Another way to activate such inhibitors is to bind them to polymers or nanostructures to create multiple binding sites.^3^, ^4^ The modification of fullerene with an iminosugar, which is an inhibitor for Jack bean α‐mannosidase, leads to 179 times higher activity of this inhibitor.^5^ Bonduelle et al. have shown that aggregates of iminosugar‐based glycopolypeptides form aggregates that increase the activity of the iminosugars as inhibitors for α‐mannosidase by a factor of 30.^6^ In both cases, the authors explained this improvement in activity by the multivalent binding character. Binding inhibitors to the backbone of polymers can also create such a scenario. Such polymer‐bound inhibitors are often used to protect drugs from degradation in the body. For example, serine protease inhibitors have been attached to poly(acrylic acid) and polysaccharides to protect drugs such as insulin from proteolysis.^2^, ^7^, ^8^

**Figure 1:**
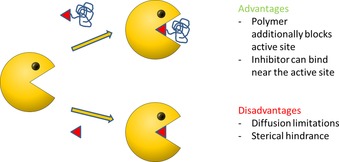
Binding concept of a competitive enzyme inhibitor attached to a polymer.

In addition to multivalent binding, the inhibitor can also be attached to the end group of a nonaggregating hydrophilic polymer. In the case of a competitive inhibitor, this would lead to the situation depicted in Figure 1. According to this concept, the inhibitor could be activated by the fact that the polymeric tail additionally blocks the active site of the enzyme. Also, the inhibitor can bind near the active site and would still be active due to its bulky tail. This might increase the variability of a potential enzyme inhibitor. On the contrary, the polymer tail might hinder binding to the active site due to steric hindrance and it will also induce diffusion limitations.

In contrast to poly(ethylene glycol) (PEG), poly(2‐oxazoline)s (POxs) interact with certain enzymes, to some extent. For example, POx–enzyme conjugates based on horseradish peroxidase (HRP) and laccase are practically inactive in water,^9^ but are highly activated in organic solvents, similar to the respective artificial enzymes.^10^ Other enzymes are less affected by POx upon conjugation.^11^ As shown by Saegusa et al., catalase can be conjugated with poly(2‐methyloxazoline) (PMOx) and poly(2‐ethyl‐2‐oxazoline) (PEtOx) nearly completely retains its activity in water.^12^ Another study reports on the conjugation of superoxide dismutase with amphiphilic POx‐based block copolymers.^13^ Here, the enzyme retained only 30 to 50 % of its original activity. Mero et al. showed that the conjugation of trypsin with PEtOx led to enzymes that showed high activity for small substrates, but a reduced activity for larger substrates.^14^ Interestingly, enzymes are fully active in POx‐based networks.^15^

There are studies that show the potential of POx derivatives as inhibitors. For example, human matrix metalloproteinases (MMPs), such as collagenase, are inhibited by telechelic POx terminated with *N*,*N*‐dimethyldodecylammonium (DDA) as end groups for use in dental adhesives.^16^ The two antibiotics ciprofloxacin and penicillin, which are both enzyme inhibitors, were shown to be active as end groups of POx.^17^ POx with a 2,2′‐iminodiacetate (IDA) end group was previously reported to diminish the activity of HRP as an entropically driven noncompetitive inhibitor.^18^ This is remarkable because IDA is not an inhibitor of HRP. The interaction of these POx‐IDA species with proteins is so strong that they form noncovalent, organosoluble conjugates with the latter.^19^

Herein, we show how POx‐IDAs inhibit the enzyme laccase and even stabilize this relatively fragile enzyme.

## Results and Discussion

Laccase is an important, copper‐based enzyme that is widely used in environmental bioremediation,^20^ chemical synthesis,^21^ biological bleaching,^22^ and in biosensors for the detection of oxidizing agents.^23^, ^24^ Typical inhibitors for this enzyme are several metal chelating ligands, such as diethylenetriaminepentaacetic acid,^25^ dithiothreitol (DTT),^26^ thioglycolic acid (TGA),^27^ oxalic acid,^28^ and citric acid.^28^ These inhibitors diminish the activity of laccase in a concentration range of 5 to 20 mm. IDA barely inhibits laccase and shows 20 % inhibition at 40 mm IDA. This weak inhibitor was attached to different POxs either at one end or at the two terminals. Different molecular weights and polymers (PMOx and PEtOx) were applied.

**Figure 2:**
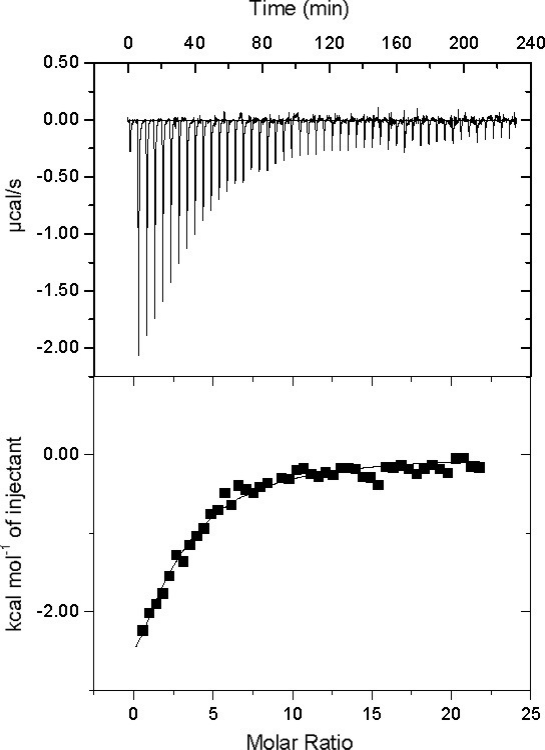
ITC isotherm of the binding interaction between PMOx_30_‐IDA (5 mm) and laccase (0.05 mm) in water at 25 °C. Each peak corresponds to the repeated injection of polymer solution (6 μL) into the reaction cell (*V*_cell_=1.34 mL) containing an aqueous solution of 0.05 mmol L^−1^ laccase. (The heat of dilution of the polymers was determined in a separate experiment and subtracted from this data.) The upper panel shows the ITC binding isotherm as power [μcal s^−1^] versus time [min]. The lower panel shows the resulting integrated heats of the binding process.

Initially, the binding reaction between laccase and PMOx‐IDA was investigated by means of isothermal titration calorimetry (ITC; Figure 2). In contrast to the interaction with HRP shown in a previous study,^18^ the reaction between laccase and PMOx_30_‐IDA is an exothermic process. This indicates a strong binding affinity of the polymer to the enzyme. Only a weak signal, and thus, no binding could be observed upon adding PMOx without the IDA end group to the enzyme; this indicates that the binding between laccase and PMOx_30_‐IDA is driven by the IDA end group. Additionally, the titration curve allows the calculation of the binding constant (0.12 mm), presuming that PMOx‐IDA and the enzyme form a 1:1 complex.

The inhibitory effect of POx‐IDA was explored on the oxidation of [2,2‐azino‐bis(3‐ethylbenzothiazoline‐6‐sulfonic acid)] diammonium salt (ABTS) by oxygen catalyzed by laccase in the presence of various concentrations of the polymer. It was found that PMOx_30_‐IDA inhibited more than 20 % of the laccase activity at a concentration of 1.25 mm. This is a 30 times lower concentration than that of free IDA to achieve the same effect. Thus, conjugation of the polymer PMOx and IDA leads to a great activation of the latter; this indicates that the general concept for polymeric inhibition, as proposed in Figure 1, seems to be valid for this system.

**Figure 3:**
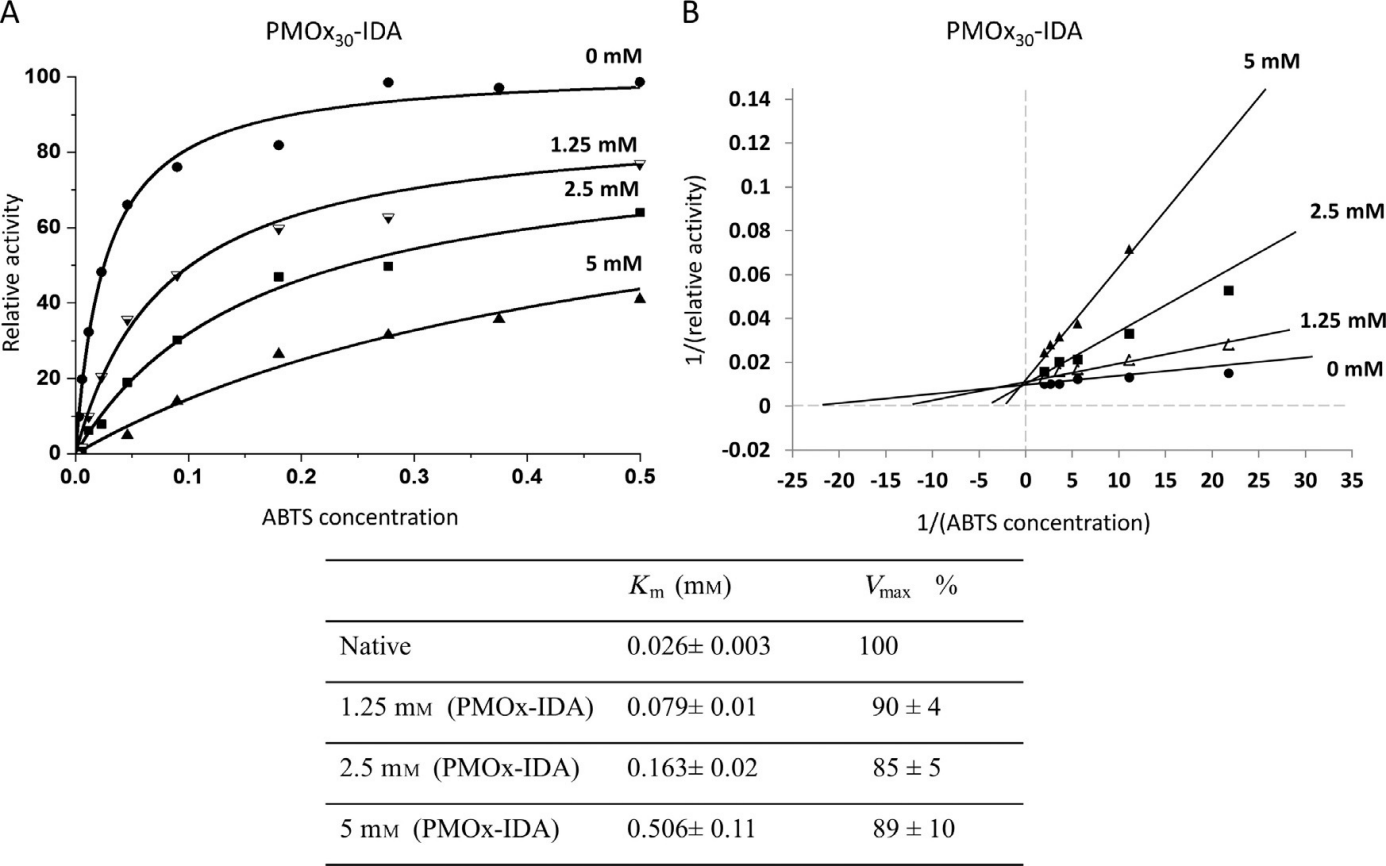
A) Michaelis–Menten plots and B) the corresponding Lineweaver–Burk plots of the activity of laccase from *Trametes versicolor* in the presence of PMOx_30_‐IDA (0, 1.25, 2.5 and 5 mm). The errors are uncertainties obtained by fitting the Michaelis–Menten equation to the data points.

To study the type of inhibition caused by PMOx‐IDA, the Michaelis–Menten parameters for the enzyme reaction in the presence and absence of CH_3_‐PMOx_30_‐IDA and IDA‐PMOx_35_‐IDA were determined. The Michaelis–Menten model was successfully used in a previous study of these polymers as inhibitors for HRP, showing noncompetitive inhibition. The kinetic experiments for laccase in this work were performed by varying the concentrations of both PMOx and ABTS. Calculation of the apparent parameters (Vappmax and Kappm ) was realized by fitting of Michaelis–Menten plots (Figure 3). The concentration of PMOx‐IDA was varied from 0 to 5 mm.

**Figure 4:**
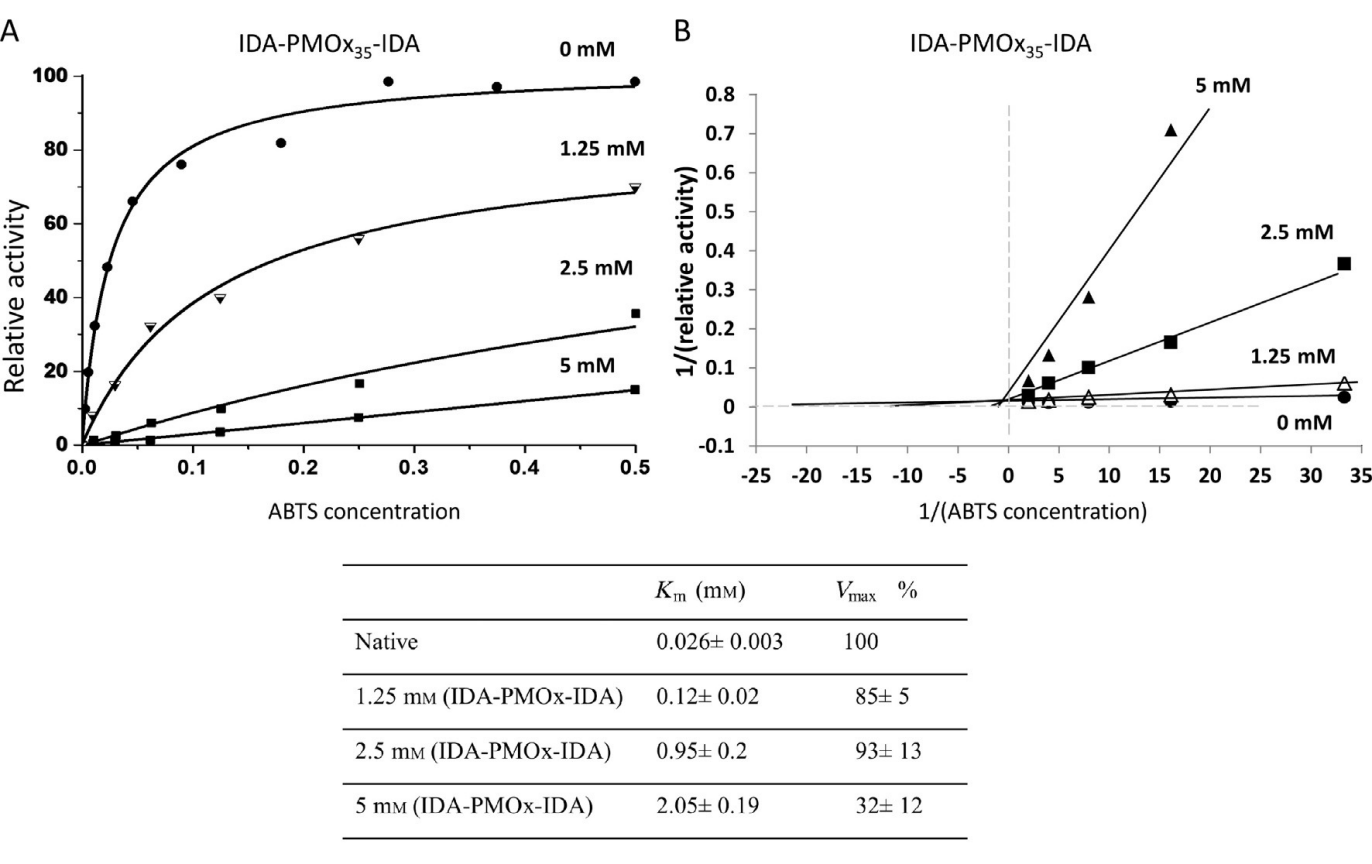
A) Michaelis–Menten plots and B) the corresponding Lineweaver–Burk plots of the activity of laccase from *T. versicolor* in the presence of IDA‐PMOx_35_‐IDA (0, 1.25, 2.5, and 5 mm). The errors are uncertainties obtained by fitting the Michaelis–Menten equation to the data points.

The Michaelis–Menten plots reveal that the increase in PMOx_30_‐IDA concentration increases the Michaelis constant, *K*_m_, from 0.026 mm of the native enzyme to 0.5 mm at 5 mm of PMOx_30_‐IDA, whereas no significant change in the maximum oxidation rate, *V*_max_, occurs. This is typical for a competitive inhibition mechanism as a major mechanism for the singly functionalized PMOx. The Lineweaver–Burk plots (Figure 3 B) clearly confirmed that the competitive inhibition mechanism given in Figures 1 and 3 can describe the inhibition of the laccase by PMOx‐IDA polymers. The inhibition kinetics of the telechelic IDA‐PMOx_35_‐IDA are shown in Figure 4.

As observed in Figure 4, IDA‐PMOx‐IDA concentrations of 1.25 and 2.5 mm afford competitive inhibition that leads to increased apparent *K*_m_ values, whereas *V*_max_ is not affected. Further increasing the polymer concentration to 5 mm affords a lower apparent *V*_max_ value and a higher apparent *K*_m_ value. This could indicate a different inhibition mechanism. Rosenfeld and Sultatos reported that the kinetics of the inhibition, in some cases, changed with concentration, which suggested that the inhibitor could be binding to a secondary binding site outside the active side of the enzyme. This can lead to apparent activation of the enzyme and in other cases to further inhibition.^29^

**Figure 5:**
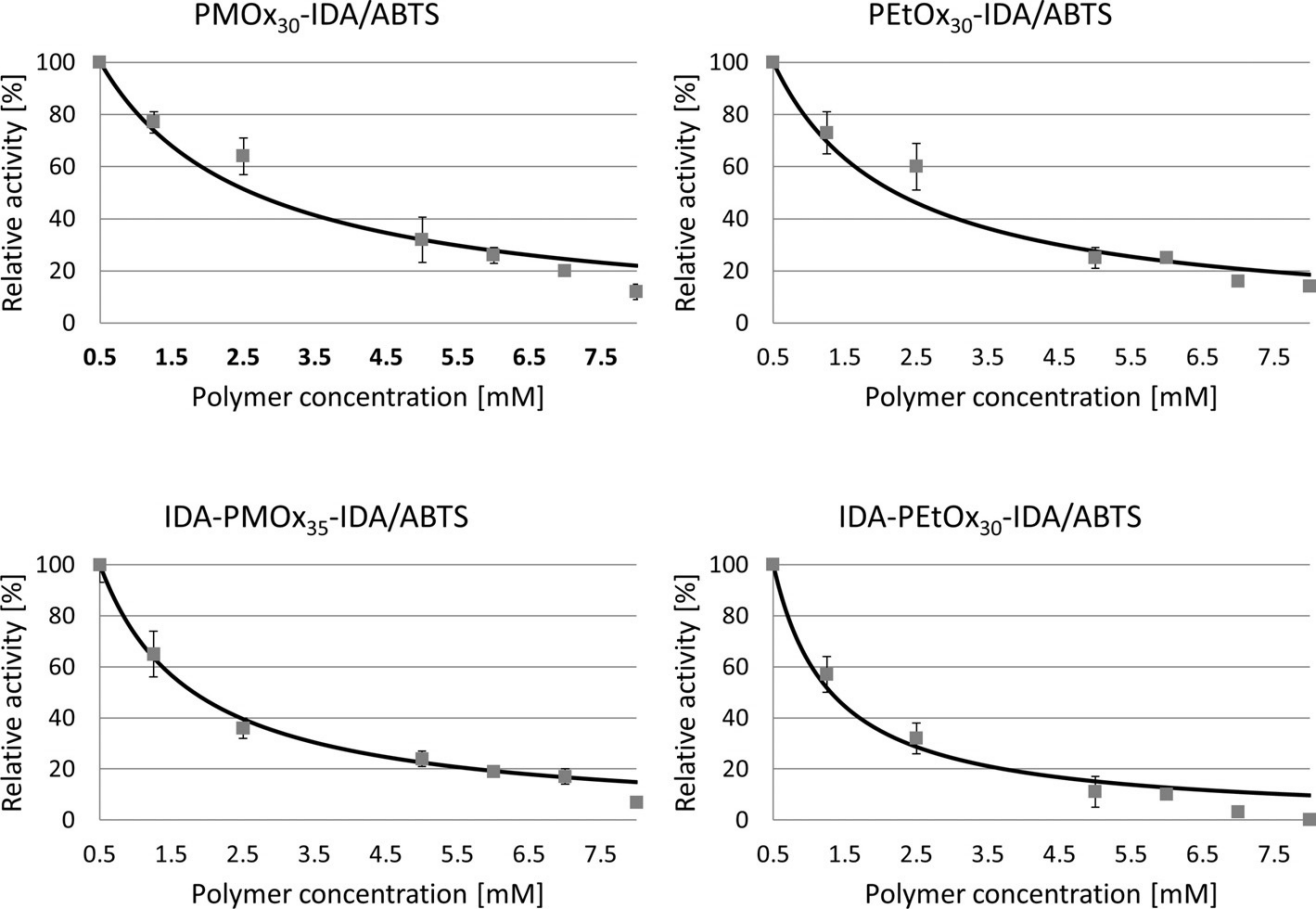
IC_50_ fitting curve of one‐sided and telechelic POx terminated with IDA against laccase from *T. versicolor*. Determination of the inhibition curves [fitted to Eq. (1)] and IC_50_ values of the polymers was performed by using OriginLab 2018b and Excel 2010 software. The laccase inhibition was measured with ABTS as a substrate (0.5 mm) at pH 4.5 in acetate buffer. All measurements were performed in triplicate, and the error bars indicate standard deviation.

The inhibition of laccase in the presence of one‐sided and telechelic PMOx and PEtOx was taken further up to 8 mm POx to determine IC_50_ values and the inhibition constant. The inhibition curves were fitted with Equation (1), which describes a competitive enzyme inhibition:

(1) $$v=\frac{V{}_{\max}\left[S\right]}{\left[S\right]+K{}_{m}\left(1+\left[I\right]/K{}_{i}\right)}$$

in which *V*_max_ and *K*_m_ are the kinetic parameters of the free enzyme, and *K*_i_ is the competitive inhibition constant. The inhibition constants *K*_i_ and the IC_50_ values were determined after fitting the inhibition values to the competitive mechanism. Figure 5 shows a graphic representation of four examples for PMOx and PEtOx with one‐side‐ and telechelic‐terminated IDA.

**Figure 6:**
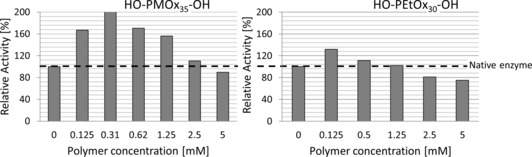
Activity profile of laccase in the presence of PMOx_35_ (left) and PEtOx_30_ (right), without IDA end groups. The laccase activity was measured with ABTS as a substrate (0.5 mm) at pH 4.5 in acetate buffer.

In contrast to typical inhibition curves, the inhibitor does not work up to a concentration of >0.5 mm in all cases. This can be explained by the fact that POx without an end group can activate laccase at low concentrations (Figure 6). The activation effect is more pronounced for PMOx than that for PEtOx.

To eliminate this effect, the inhibition curves were fitted with a concentration of 0.5 mm as a starting value. As observed in Figure 5, the curves are well fitted to the competitive mechanism. The inhibition constants *K*_i_ are in the range of 0.04 to 0.15 mm. Closer inspection of the inhibition curves reveals that, in most cases, the curve does not fit the inhibition rates at inhibitor concentrations of 7 mm and higher. This is probably due to a different inhibition mechanism at higher concentrations, which has been reported for low‐molecular‐weight, competitive inhibitors.^30^ In the case of IDA‐PEtOx_30_‐IDA, the activity can be inhibited by more than 99 %, which makes this polymer a dead‐end inhibitor. The results of *K*_i_ and IC_50_ values for all polymers, calculated from the respective curves, are presented in Table 1.

**Table 1:** *K*_i_ constants and IC_50_ values of various one‐sided and telechelic POx‐IDA as inhibitors for laccase with ABTS as a substrate.

	IC_50_ [mm]	*K*_i_ [mm]	*R* ^2^
**One‐sided polymers**			
Me‐PMOx_21_‐IDA	2.2±0.33	0.04	0.081
Me‐PMOx_30_‐IDA	2.6±0.11	0.08	0.92
Me‐PEtOx_17_‐IDA	1.6±0.05	0.03	0.97
Me‐EtOx_30_‐IDA	2.6±0.07	0.06	0.93
**Telechelic polymers**			
IDA‐PMOx_9_‐IDA	3.9±0.06	0.15	0.93
IDA‐PMOx_35_‐IDA	1.7±0.07	0.04	0.97
IDA‐EtOx_13_‐IDA	2.9±0.06	0.10	0.90
IDA‐EtOx_30_‐IDA	1.3±0.02	0.04	0.94

As observed from the IC_50_ values in Table 1, all polymers are inhibitors for laccase and are generally more active than that of IDA alone. The IC_50_ values are in a range of 1.3 to 3.9 mm. Thus, the activation factor for IDA attached to POx is between 30 and 60. Furthermore, it can be seen that the telechelic polymers are up to two times more active than the one‐side‐terminated analogues. This could be interpreted as an effect caused by multiple binding at the protein. This is in contrast to the results found for HRP, for which telechelic termination had no further effect on the inhibition potency.^18^

Moreover, the dependence of activity on the molecular weight of the polymers was investigated with one‐ and two‐side‐terminated IDA polymers with two different molecular weights. The telechelic macromolecules with high molecular weight are also up to two times more active than that of the smaller ones because the larger polymer tail would result in a stronger blocking of the active site. Additionally, upon comparing the inhibition of PMOx and PEtOx derivatives with similar lengths, the IC_50_ and *K*_i_ values are almost identical, that is, the hydrophilicity of the polymers is not the major driving force, which is most likely to be the end group in combination with the bulky tail. The respective polymers without specific end groups show a weak inhibition of the enzyme at higher concentrations. In contrast, one‐sided POx‐IDA with lower molecular weights are more than two times more active inhibitors than that of the respective higher molecular weight polymers. This is possibly because the affinity to the active side of the enzyme is higher for the low‐molecular‐weight polymers due to lower steric hindrance.

**Figure 7:**
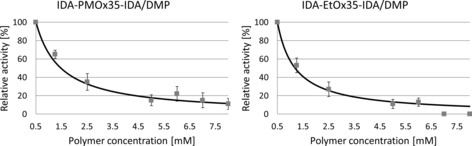
IC_50_ fitting curves of IDA‐POx‐IDA versus laccase from *T. versicolor* by using 2.8 mm DMP as a substrate at pH 4.5 in acetate buffer. The inhibition curves were fitted according to Equation (1) by using the fitting tool of OriginLab 2018b. All measurements were performed in triplicate, and the error bars indicate standard deviation.

To broaden the validity of the concept, 2,6‐dimethoxyphenol (DMP) was used as a second substrate and inhibition in the presence of IDA‐PMOx_35_‐IDA and IDA‐PEtOx_30_‐IDA (0.5–8 mm) was investigated (Figure 7).

As observed in Figure 7, the IC_50_ curves observed with DMP as substrate look similar to those found with ABTS as substrate. *K*_i_ and IC_50_ values for the two polymers were calculated from the respective curves and are listed in Table 2. These data show that the inhibition of the polymers for laccase with DMP as a substrate is stronger, as suggested by the tenfold lower *K*_i_ values. This is expected for competitive inhibition because DMP has a lower affinity to the active site of the enzyme than that of ABTS (*K*_m ABTS_=0.026, *K*_m DMP_=0.037).

**Table 2:** *K*_i_ and IC_50_ values for IDA‐PMOx_35_‐IDA and IDA‐PEtOx_30_‐IDA as inhibitors for laccase with DMP as substrate. Fitting was performed according to Equation (1) with an inhibitor concentration of 0.5 mm as the starting point and *K*_m_=0.037 mm determined from Michaelis–Menten kinetics with DMP as a substrate.

	IC_50_ [mm]	*K*_i_ [mm]	*R* ^2^
IDA‐PMOx_35_‐IDA	1.9±0.045	0.003	0.96
IDA‐EtOx_30_‐IDA	1.1±0.035	0.002	0.99

**Figure 8:**
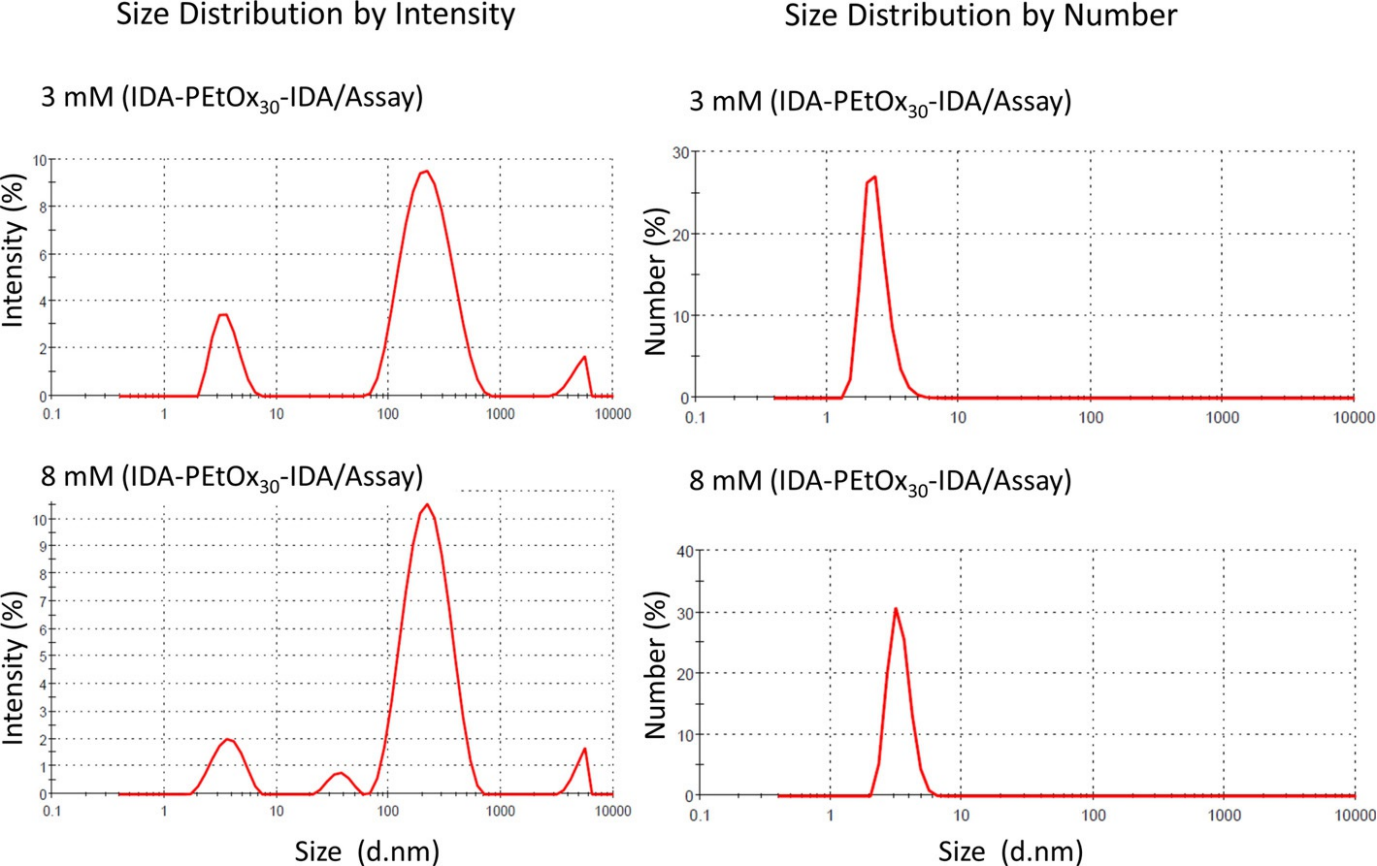
DLS measurements (intensity plot left, number plot right) of laccase DMP assay buffer containing IDA‐PEtOx30‐IDA at 3 (top) and 8 mm (bottom).

Interestingly, the inhibitor IDA‐EtOx_30_‐IDA can practically fully inhibit laccase at a concentration of 7–8 mm. This is unusual, although not unique, for competitive inhibitors. To explore if this effect might derive from certain superstructures formed at higher concentrations, dynamic light scattering (DLS) experiments were performed in the respective assay buffer solution at concentrations of 3 and 8 mm, respectively. Our hypothesis was that the POx‐IDA might form aggregates at higher concentrations, which would then act as multivalent inhibitors. The latter are known sometimes to activate the attached inhibitor structures by up to 179 times.^5^ Such an effect might explain a seemingly increasing activity of POx‐IDA at higher concentrations. As observed in the intensity plots shown in Figure 8, solutions at both concentrations show a peak at 2.5 nm, which can be attributed to the single polymer chains, and a peak at 250 nm, which most likely originates from unspecific aggregates. The only difference is a peak at 28 nm, which only occurs at higher concentration. This peak could be an aggregate, which might indeed be responsible for the full inhibition of laccase. The intensity of this peak is very low, resulting in less than 0.01 % of all molecules in the number plot of the DLS curve (Figure 8, right). Thus, it seems unlikely that this aggregate is responsible for the higher activity.

To investigate if the POx‐IDA inhibitors for laccase are reversible, laccase solutions that contained 20 mm of IDA‐PMOX_35_‐IDA and IDA‐PEtOx_30_‐IDA were prepared. Then, different volumes of these solutions were added to the ABTS assay and the resulting activity was compared with that found for a laccase solution without polymer.

**Figure 9:**
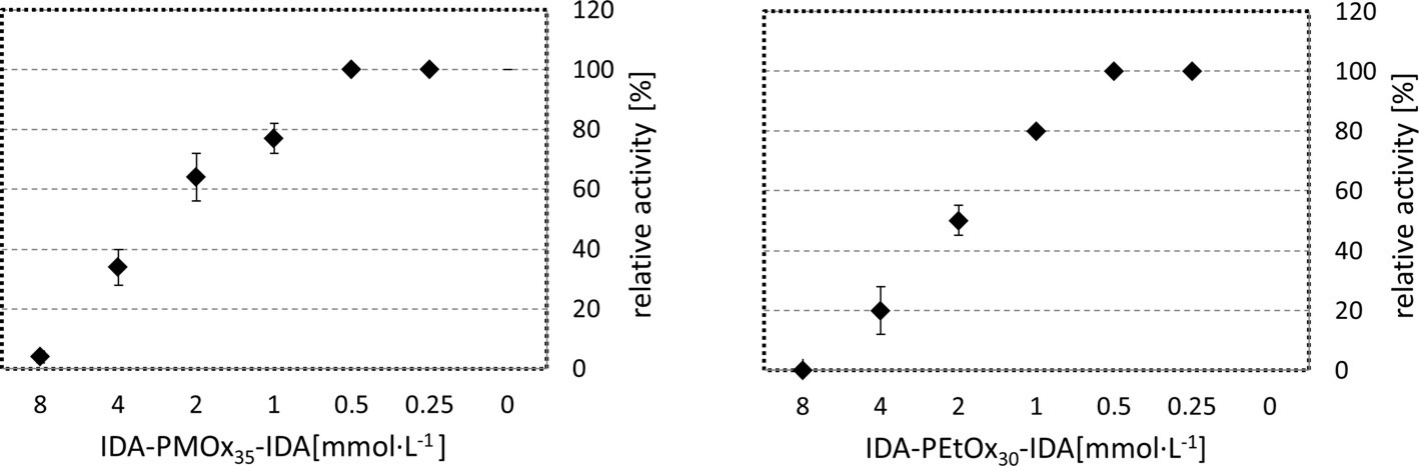
Laccase activity after diluting a stock solution of laccase incubated with 20 mm POx‐IDA in relation to the same solution without inhibitor. Stock solution: laccase (2.2×10^−3^ mg mL^−1^) in 100 mm acetate buffer with 20 mm POx‐IDA. Procedure: different volumes of the stock solution (400, 200, 100, 50, 25, and 12.5 μL) filled up to 1 mL with the standard laccase ABTS assay. The activity was determined spectrophotometrically at *λ*=420 nm and compared with the activity of a respective aqueous, buffered laccase solution.

As observed in Figure 9, the relative enzyme activity increases with greater dilution and reaches its original native activity at a concentration of 0.5 mm polymer in the assay solution. This is in agreement with the inhibition curves shown in Figure 5, which confirms that the inhibition of laccase with the polymers described herein is fully reversible and that the activity is fully preserved after one week of storage.

Laccase is a rather fragile enzyme, which quickly loses its activity during storage, particularly in aqueous solution. Several stabilizers were used for this enzyme, but, so far, only immobilization and covalent crosslinking led to greatly improve storage stability.^31^, ^32^, ^33^, ^34^ To investigate if POx‐IDA was not only inhibiting, but also stabilizing laccase, solutions (100 mm acetate buffer, pH 5, 2.2×10^−3^ mg mL^−1^) of the enzyme containing various POx at different concentrations were prepared and stored at room temperature for up to 28 days. The concentration of POx was set to 5, 10, and 20 mm in the incubated solutions. The activity was measured after different intervals of storage at room temperature by adding 25 μL of the stock solution to 0.975 mL laccase assay, which resulted in a POx concentration ≤0.5 mm.

**Figure 10:**
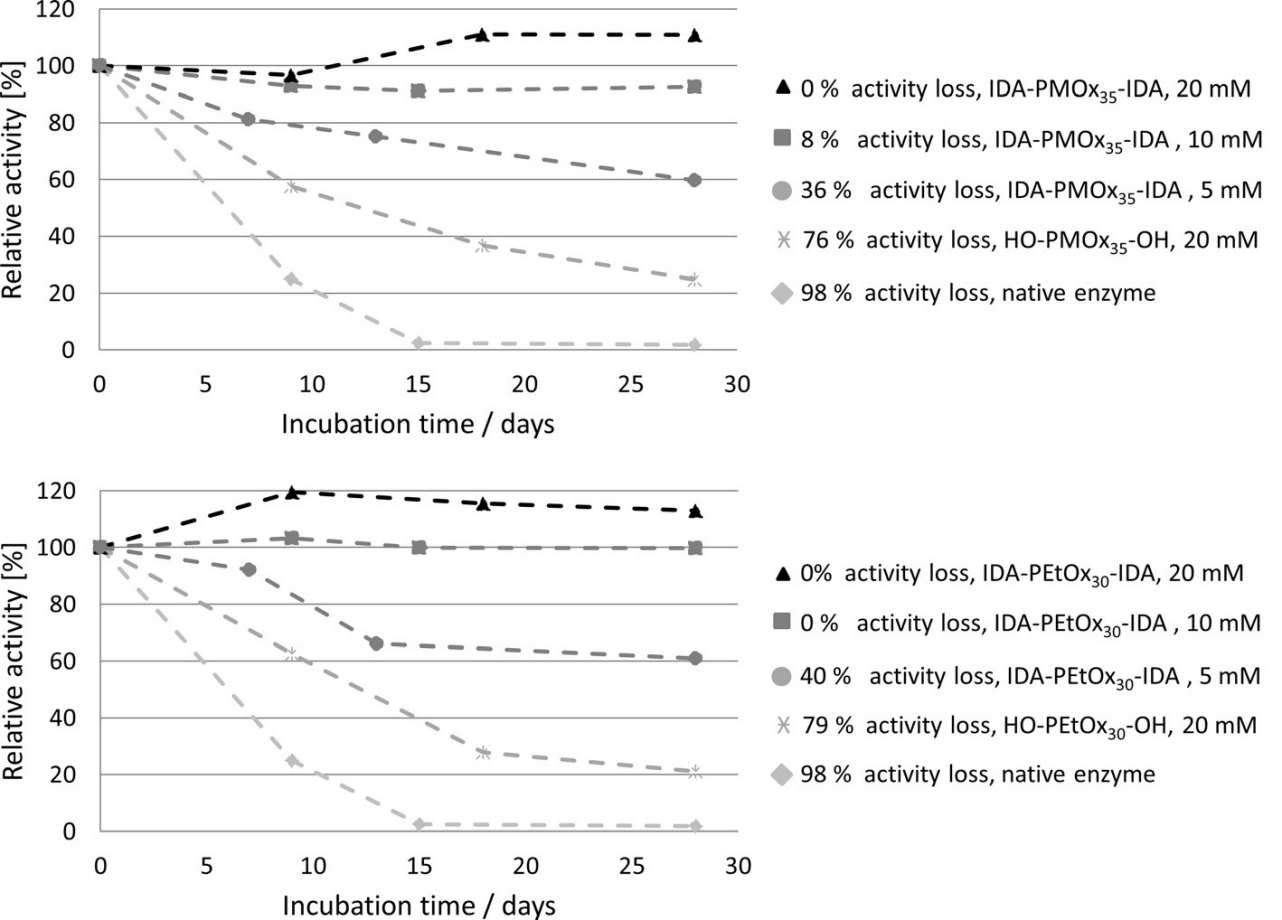
Stability effect of 5, 10, and 20 mm POx‐IDA on laccase activity in acetate buffer, pH 4.5, for 28 days.

As observed in Figure 10, laccase in water becomes practically inactive after 18 days of storage. POx without an end group already stabilizes laccase. The presence of 20 mm PMOx and PEtOx resulted in a retention of 20 % of activity after 28 days of storage. Upon adding 5 mm IDA‐PMOx_35_‐IDA and IDA‐PEtOx_30_‐IDA, the activity after 28 days was still about 60 %, showing the role of the IDA end groups. If the polymer concentration was increased from 5 to 10 mm, an increase in stability was observed. Laccase in the presence of 10 mm IDA‐PMOx_35_‐IDA retained 92 % of the laccase activity after 28 days. IDA‐PMOx_30_‐IDA completely protected the enzyme for this period of time. Further increasing the POx‐IDA concentration to 20 mm resulted in full protection of laccase in both cases.

**Figure 11:**
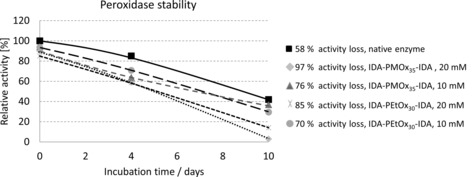
Stability effect of 10 and 20 mm POx‐IDA on HRP activity in 100 mm phosphate‐citrate buffer at pH 5 for 10 days.

Thus, POx‐IDAs stabilize the enzyme in its deactivated state. In contrast to laccase, HRP is not stabilized by POx‐IDAs (Figure 11). We hypothesize that this is due to a different inhibition mechanism. As shown previously, POx‐IDA is a noncompetitive inhibitor for HRP. This could be expected because the stability mechanisms are not universal and must be explored for each protein separately.^35^, ^36^

## Conclusion

We showed that POxs with IDA end groups were competitive inhibitors for laccase and act as stabilizers for this enzyme. This supports the concept illustrated in Figure 1 that inhibitors bound to hydrophilic polymers as end groups are initially activated and the bulky polymeric tail additionally blocks the active site of an enzyme. The behavior of POx‐IDA towards laccase is essentially the opposite of that towards HRP. POx‐IDA are noncompetitive inhibitors for HRP and do not stabilize this enzyme. Thus, polymers with enzyme inhibitors as end groups are a versatile and interesting way to bring to functions to these relevant drugs.

## Experimental Section

**Instruments**: ^1^H NMR spectra were recorded in CDCl_3_ by using a Nanobay AVANCE‐III HD‐400 spectrometer with a 5 mm BBFOsmart probe from Bruker BioSpin GmbH operating at 400 MHz, and on a DD2‐500 spectrometer with a 5 mm triple resonance H(C,X) probe from Agilent Technologies operating at 500 MHz. UV/Vis spectroscopy was performed on an Analytik Jena Specord 210 spectrophotometer with a double‐beam photometer to monitor the enzyme activity. Size‐exclusion chromatography (SEC) was performed on a Viscotek GPCMax instrument equipped with a refractive index (RI) detector (tempered to 55 °C) by using a Tosoh TSKgel GMHHR‐M (5.0 μm pores, 2×+1× precolumn) column set. As an eluent, saline *N*,*N*‐dimethylformamide (DMF+LiBr, 20 mmol) was used at 60 °C at a flow rate of 0.70 mL min^−1^. Calibration was performed with polystyrene standards (Viscotek). ITC was performed on a MicroCal VP‐ITC instrument that measured heat evolved or absorbed in liquid samples as a result of mixing precise amounts of reactants. DLS measurements were performed on a Malvern Zetasizer Nano S (ZEN 1600) instrument in aqueous buffer at 25 °C and polymer concentrations of 3 and 8 mm. All polymerizations were performed by using a microwave‐assisted synthesizer from CEM with a vertically focused IR sensor.

**Materials**: All chemicals and solvents were purchased from Acros, Merck, Fluka, and Sigma Aldrich. HRP (EC 1.11.1.7) and laccase from *T. versicolor* were purchased from Sigma Aldrich. DMP was purchased from Acros. ABTS was purchased from Sigma–Aldrich.

**Synthesis of POx‐IDA**: The syntheses of the polymers terminated with IDA were performed according to procedures reported in the literature. The composition of the polymers was calculated from ^1^H NMR spectra in CDCl_3_.^18^ Analytical data for the resulting polymers are given in Table 3.

**Table 3:** Analytical data of the different polymers determined by SEC and ^1^H NMR spectroscopy measurements.^[a]^

Polymer	*M*_n, NMR_ [kg mol^−1^]	F^d[b]^ [%]	*M*_n, SEC_ [kg mol^−1^]	PDI^[b]^
CH_3_‐P(MOx_21_)‐IDA	2.0	100	2.8	1.1
CH_3_‐P(MOx_30_)‐IDA	2.7	100	2.8	1.2
IDA‐P(MOx_9_)‐IDA	1.1	99	1.0	1.3
IDA‐P(MOx_35_)‐IDA	2.7	100	3.1	1.3
CH_3_‐P(EtOx_17_)‐IDA	2.1	85	1.4	1.2
CH_3_‐P(EtOx_30_)‐IDA	3.1	100	2.8	1.1
IDA‐P(EtOx_13_)‐IDA	1.6	90	1.0	1.1
IDA‐P(EtOx_30_)‐IDA	3.1	94	3.4	1.1

[a] The initiator for IDA‐POx‐IDA was 1,4‐dibromobut‐2‐ene (DBB) and the initiator for CH_3_‐POx‐IDA was methyl tosylate. Termination was performed with 2.5 equivalents of dimethyl 2,2′‐iminodiacetate for CH_3_‐POx‐IDA and 5 equivalents for IDA‐POx‐IDA. [b] Degree of functionality. [c] PDI: polydispersity index.

**Laccase assay with ABTS substrate**: The activity of pure laccase from *T. versicolor* was determined according to a Majcherczyk modified assay with 0.5 mm ABTS as a color‐generating substrate in 100 mm acetate buffer at pH 4.5.^37^ Coloration was monitored at a wavelength of 420 nm at 25 °C by using a spectrophotometer (Analytik Jena AG, Jena Germany). Different concentrations of POx (in the range from 0.5 to 8 mm) were dissolved in ABTS solution (900 μL). Then, laccase (100 μL, 0.05 mg mL^−1^, about 0.8 μm) was mixed with the aqueous, buffered ABTS polymer mixture and the increase in absorbance was measured for 5 min. The molar extinction coefficient of oxidized ABTS is 36.6 m ^−1^ cm^−1^.

**Laccase assay with DMP substrate**: The laccase activity was determined according to a method reported by Paszczyński et al. by using 2.8 mm DMP substrate in 100 mm acetate buffer pH 4.5.^38^ The reaction mixture was prepared analogously to that for the ABTS assay and the increase in absorbance was photometrically determined at a wavelength of 468 nm for 5 min. The molar extinction coefficient of oxidized DMP is 49.6 mm ^−1^ cm^−1^.

**Storage stability of laccase**: The stability of the enzyme was tested by incubating 1 mL of the enzyme (2.2×10 ^−3^ mg mL^−1^) and polymer at different concentrations (5, 10, 20 mm) for 28 days in acetate buffer at pH 4.5. The activity of the incubated enzyme was then determined at different time points as follows: the polymer enzyme solution (25 μL) was added to the ABTS assay solution (1 mL) at 25 °C. The activity was compared with the initial activity of laccase at the beginning of the measurement.

**Storage stability of HRP**: The stability of HRP was tested by incubating the enzyme (1 mL, 1.25×10^−3^ mg mL^−1^) and polymer at concentrations of 10 and 20 mm, for 20 days in 0.2 m phosphate/0.1 m citrate buffer at pH 5. The activity of the incubated enzyme was then determined at different time points as follows: the polymer enzyme solution (25 μL) was mixed with the ABTS buffer solution (1425 μL, 0.2 m phosphate/0.1 citrate buffer at pH 5 and 5 mm of ABTS) then hydrogen peroxide solution (50 μL, 0.3 wt %) was added and the increase in absorbance was photometrically determined at 25 °C at a wavelength of 405 nm. The activity was compared with the initial activity of HRP at the beginning of the measurement.

## Conflict of interest

*The authors declare no conflict of interest*.
